# Whole-genome resequencing and bisulfite sequencing provide new insights into the feeding habit domestication in mandarin fish (*Siniperca chuatsi*)

**DOI:** 10.3389/fgene.2022.1088081

**Published:** 2023-01-12

**Authors:** Ling Li, Shan He, Ming-Hui Lin, Yan-Peng Zhang, Heiner Kuhl, Xu-Fang Liang

**Affiliations:** ^1^ Chinese Perch Research Center, College of Fisheries, Huazhong Agricultural University, Wuhan, China; ^2^ Engineering Research Center of Green Development for Conventional Aquatic Biological Industry in the Yangtze River Economic Belt, Ministry of Education, Wuhan, China; ^3^ Department of Ecophysiology and Aquaculture, Leibniz-Institute of Freshwater Ecology and Inland Fisheries, Berlin, Germany

**Keywords:** Mandarin fish, resequencing, whole-genome bisulfite sequencing, feeding habit domestication, selective sweep

## Abstract

Mandarin fish (*Siniperca chuatsi*) is one of the most economically important fish in China. However, it has the peculiar feeding habit that it feeds solely on live prey fish since first-feeding, while refuses dead prey fish or artificial diets. After the specific training procedure, partial individuals could accept dead prey fish and artificial diets. The genetic basis of individual difference in artificial diet feeding habit is still unknown. In the present study, the resequencing was performed between 10 individuals which could be domesticated to accept artificial diets and 10 individuals which could not. Through the selective sweep analysis based on heterozygosity (*Hp*) and population differentiation coefficient (*Fst*), 57 candidate windows were identified as the putative selected regions for feeding habit domestication of mandarin fish, involved in 149 genes. These genes were related to memory, vision and olfaction function, which could be potential targets of molecular marker assistant breeding of artificial diet feeding trait. Beside of the DNA sequence, we also explored the potential role of DNA methylation in feeding habit domestication in mandarin fish. Whole-genome bisulfite sequencing was performed between the individuals which could be domesticated to accept artificial diets and those could not. 5,976 differentially methylated regions were identified, referring to 3,522 genes, such as the genes involved in cAMP signaling pathway. The DNA methylation changes of these genes might contribute to the adaption of artificial diets in mandarin fish. In conclusion, the putative selected regions and the differentially methylated regions were identified in the whole genome, providing new insights into the feeding habit domestication from live prey fish to artificial diets in mandarin fish. And the involved genes were identified as the candidate genes for molecular breeding of artificial diet utilization in mandarin fish.

## Introduction

Mandarin fish (*Siniperca chuatsi*) is one of the most economically important fish in China. The annual yield has been over 300,000 tonnes, and the output value has been more than three billion US dollars since 2015 (FAO). However, due to its special feeding habit of live prey fish (Liang et al., 2001; Liang et al., 2008), the stable supply of palatable live prey fish resulted in high farming cost for mandarin fish. After the specific training procedure (Liang et al., 2001), some individuals could accept dead prey fish and artificial diets, but there are still some individuals could not. The genetic basis of individual difference in artificial diet feeding habit is still unknown.

In our previous studies, we have identified several single nucleotide polymorphisms (SNPs) in functional genes through PCR product sequencing. One SNP was identified in the appetite regulatory gene *npy* (Sun et al., 2015) and two SNPs were identified in the digestive enzyme gene pepsinogen (*pep*) (Yi et al., 2013) related to dead prey fish feeding trait, two SNPs were identified in the learning and memory gene protein phosphatase 1 (*pp1*) related to artificial diet feeding trait (Cheng et al., 2015). Whole-genome resequencing has been applied in fish to identify candidate genes associated with traits of biological and commercial interest, such as genes associated with growth, early development and immunity traits in Nile tilapia (Cádiz et al., 2020), genes associated with body color and fin morphology in Siamese fighting fish (Kwon et al., 2022). However, the large-scale screening of selected genes and molecular markers associated with feeding habit domestication trait from the whole genome is in urgent need to research.

DNA methylation as one of the most studied epigenetic modifications, has been reported to play important roles in fish, including feeding (Cai et al., 2018; Dou et al., 2018), metabolism (Cai et al., 2020; Kumkhong et al., 2020; Zhu et al., 2020), growth (Zhong et al., 2014; Si et al., 2016; Huang et al., 2018), sex determination and gonadal differentiation (Navarro-Martín et al., 2011; Shao et al., 2014; He et al., 2020a), and environmental adaptation (Morán et al., 2013; Pierron et al., 2014; Veron et al., 2018). During the feeding habit transition from carnivory to herbivory in grass carp, the DNA methylation level of umami receptor *t1r1* increased and the mRNA expression level decreased (Cai et al., 2018). In our previous study, we found that DNA methylation could regulate the mRNA expression of *t1r1*, further contributed to the feeding habit domestication from live prey fish to dead prey fish in mandarin fish (Dou et al., 2018). Whole-genome DNA methylation patterns have been characterized in fish, and differential methylation analyses identified key genes related to feeding, such as genes involved in diet response in Nile tilapia (Podgorniak et al., 2022), and genes involved in foraging in grass carp (Li et al., 2021). However, little is known about the whole-genome DNA methylation assay related to the feeding habit domestication in mandarin fish.

Chromosome-level reference genome assembly of mandarin fish (He et al., 2020b) allowed us to identify genome-wide variants and DNA methylation changes between mandarin fish with different performance in accepting artificial diets. In the present study, the whole-genome resequencing and bisulfite sequencing were performed between the individuals which could be domesticated to accept artificial diets and those could not. The putative selected regions and the differentially methylated regions were identified in the whole genome, and the involved genes were identified as the candidate genes for molecular breeding of artificial diet utilization in mandarin fish.

## Materials and methods

### Fish

Mandarin fish (*Siniperca chuatsi*) (Huakang No. 1) used in the present study were obtained from the Chinese Perch Research Center of Huazhong Agricultural University (Wuhan, China), and kept in tanks with continuous system of water filtration and aeration at constant temperature (25°C ± 1 C). Mandarin fish (50 ± 5 g) were trained to accept artificial diets according to our previously published training procedure (Liang et al., 2001; He et al., 2021). The composition of artificial diets was reported in our previous study (He et al., 2021). This study was approved by the Institutional Animal Care and Use Ethics Committee of Huazhong Agricultural University (Wuhan, China) (HZAUFI-2020-0020).

### Whole-genome resequencing and mapping

10 individuals which could be domesticated to accept artificial diets and 10 individuals which could not were sampled. To minimize possible suffering, the fish were anesthetized with MS-222 (200 mg/l) (Redmond, WA, United States) until loss of equilibrium. Pelvic fins were dissected, and genomic DNA was extracted following the standard phenol-chloroform extraction procedure. DNA integrity was evaluated by agarose gel electrophoresis. DNA purity was checked using the NanoPhotometer^®^ spectrophotometer (IMPLEN, CA, United States). DNA concentration was determined by Qubit^®^ 2.0 Flurometer (Life Technologies, CA, United States) with a Qubit^®^ DNA Assay Kit. Paired-end sequencing library was constructed for each of the 20 samples according to the manufacturer’s instructions (Illumina Inc., San Diego, CA, United States). The libraries were sequenced on Illumina HiSeq X Ten platform (PE150). Library construction and sequencing were performed by Tianjin Novogene Bioinformatic Technology Co., Ltd. (Tianjin, China).

The raw data was filtered to remove the adapters, reads containing more than 10% unknown bases, and reads with more than 50% low-quality bases (Phred score ≤5). Then the clean reads were aligned against our mandarin fish reference genome (version: sinChu7) (He et al., 2020b) using Burrows-Wheeler Aligner software (bwa mem -t 4 -k 32 -M) (Li and Durbin, 2009). The output SAM files were converted into BAM files, then sorted according to genome coordinates. Subsequently, PCR duplicates were removed using SAMtools software (Li et al., 2009).

### Single nucleotide polymorphism calling and annotation

SNP calling was performed using SAMtools software (Li et al., 2009). SNPs were filtered by Perl script with the parameters: read depth ≥4, missing rate <0.1, minor allele frequency (MAF) ≥ 0.05, and genotype quality score ≥10. SNPs were annotated using ANNOVAR software (Wang et al., 2010) based on the GFF3 file of our mandarin fish reference genome. Synonymous and non-synonymous variants were predicted.

### Selective sweep analysis

A sliding window method (the window size of 100 kb and the step size of 50 kb) was applied for the selective sweep analysis. Heterozygosity (*Hp*) and population differentiation coefficient (*Fst*) were calculated. Windows with top 10% *Hp* values and top 10% *Fst* values were considered to be candidate selected regions. To know the biological function of genes within candidate selected regions, Gene Ontology (GO) (http://geneontology.org/) and Kyoto Encyclopedia of Genes and Genomes (KEGG) pathway (https://www.genome.jp/kegg/pathway.html) enrichment analyses were performed.

### Whole-genome bisulfite sequencing (WGBS)

According to our previous study, the mandarin fish did not eat artificial diets during domestication processes were named Group W (n = 56), and the mandarin fish ate artificial diets were named Group X (n = 24) (He et al., 2021). Three fish were randomly selected from each group, anesthetized with MS-222 (200 mg/l) and euthanized. Genomic DNA was isolated from liver tissue with a standard phenol-chloroform method. DNA integrity and purity were evaluated by agarose gel electrophoresis. DNA concentration was determined by Qubit^®^ 2.0 Flurometer with a Qubit^®^ DNA Assay Kit (Life Technologies). DNA was randomly fragmented by sonication. The fragments with an average size of 200–400 bp were purified according to the manufacturer’s instructions of Agencort AMPure XP-Medium Kit (Beckman Coulter, United States), followed by DNA-end repair, 3′-dA overhang and ligation of methylated sequencing adaptors. Then bisulfite conversion was performed with ZYMO EZ DNA Methylation-Gold Kit (Zymo Research, Irvine, CA, United States). After desalting, size selection, PCR amplification and size selection again, qualified libraries were sequenced on Illumina HiSeq X Ten platform (PE150). Library construction and sequencing were performed by Beijing Genomics Institute (BGI, Wuhan, China).

### Identification of differentially methylated regions and differentially methylated genes

WGBS reads mapping to our mandarin fish reference genome (version: sinChu7) (He et al., 2020b) (bismark --bowtie2), deduplication (deduplicate_bismark) and methylation calling (bismark_methylation_extractor --ignore 10 --ignore_r2 10) were performed with Bismark (Krueger and Andrews, 2011). Data were filtered so that only sites with read depth ≥10 were retained. Sites that were in the 99.9th percentile of coverage were also removed from the analysis to account for potential PCR bias. Differentially methylated regions were identified by methylKit (tiling windows, win. size = 200 bp, step. size = 200 bp) (Akalin et al., 2012), with the methylation difference larger than 25% and *q*-value <0.01. To know the biological function of differentially methylated genes, GO and KEGG pathway enrichment analyses were performed.

## Results

### Whole-genome resequencing and mapping

Resequencing was performed between 10 individuals which could be domesticated to accept artificial diets and 10 individuals which could not. A total of 169.45 Gb raw data was generated. After filtering, 169.29 Gb clean data was obtained. The effective rates (the ratio of clean data to raw data) were 99.81%–99.94%. The Q20 (sequencing error rate <1%) and Q30 (sequencing error rate <0.1%) were 97.03%–97.84% and 91.78%–93.64%, respectively. The GC contents were 40.64%–41.77% (Supplementary Table S1). The average sequencing depth was 8.57–10.19×. Of the clean reads, 99.65%–99.79% were mapped to our mandarin fish reference genome (version: sinChu7). 97.75% reference genome bases had at least 1 × coverage, and 92.69% had at least 4 × coverage (Supplementary Table S2).

### Variation discovery

**FIGURE 1:**
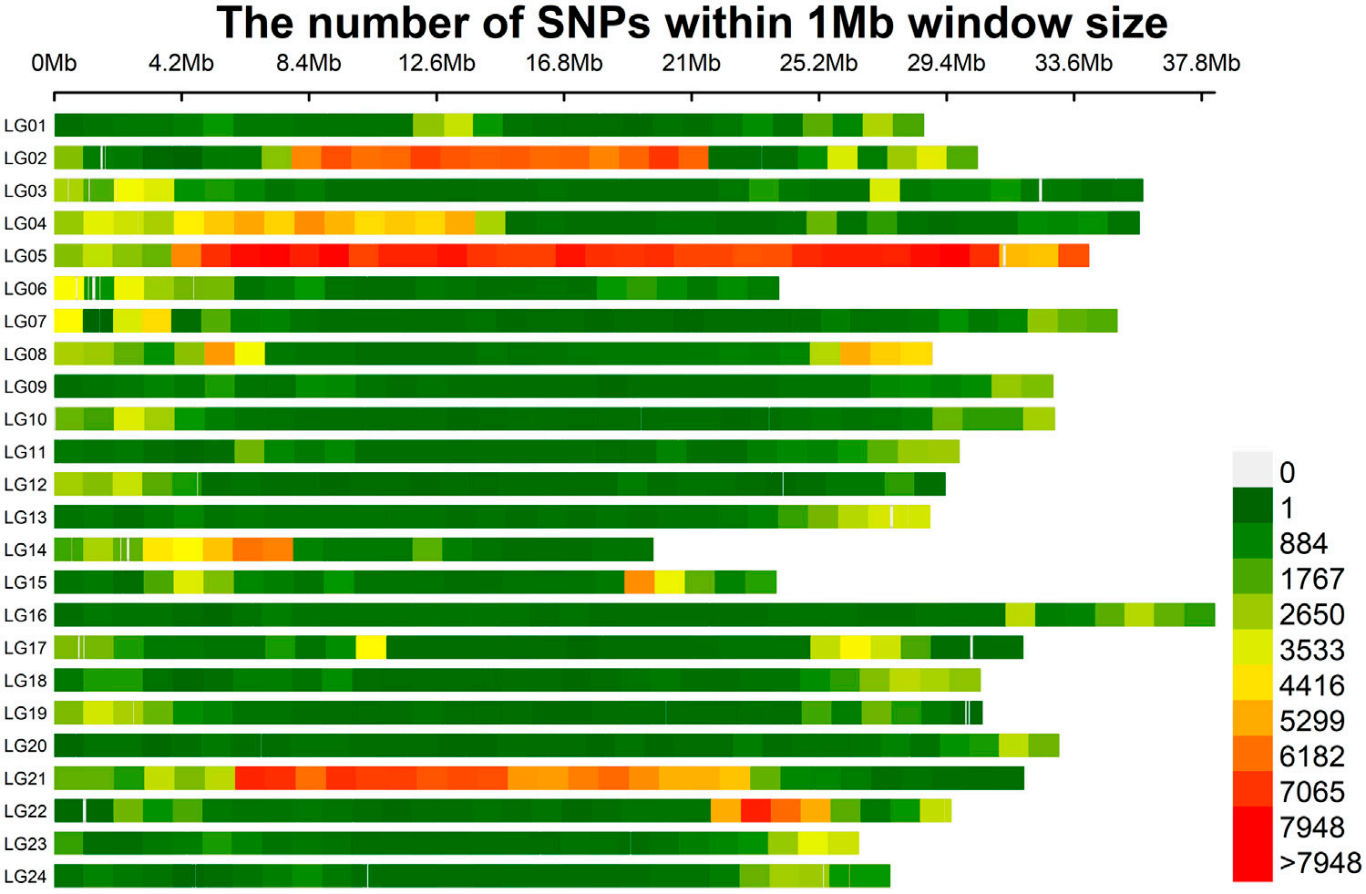
The distribution of SNPs on the chromosomes of mandarin fish genome.

A total of 1,132,712 SNPs were identified, of which 1,119,169 (98.80%) were located on 24 assembled chromosomes of mandarin fish, with the highest density on LG05 and LG21 (Figure 1). 93,684 (8.27%) SNPs were in exonic regions, 613,568 (54.17%) SNPs were in intronic regions, and 425,460 (37.56%) SNPs were in intergenic regions (Table 1). In exonic regions, 216 stop-gain SNPs, 11 stop-loss SNPs, 26,765 synonymous SNPs and 22,414 non-synonymous SNPs were identified, respectively. The ratio of transition to transversion (ts/tv) was 1.461.

**TABLE 1:** Statistics of SNP detection.

Category		Number of SNPs
Upstream		29,584
5′UTR		7,842
Exonic	Stop gain	216
	Stop loss	11
	Synonymous	26,765
	Non-synonymous	22,414
Intronic		613,436
Splicing		132
3′UTR		35,376
5′UTR/3′UTR		1,060
Downstream		26,046
Upstream/Downstream		4,347
Intergenic		365,483
ts		672,401
tv		460,311
ts/tv		1.461
Total number of SNPs		1,132,712

Note: Upstream: the variations located within 1 kb upstream of gene; Splicing: the variations located at the splicing site (near the exon/intron boundary); Downstream: the variations located within 1 kb downstream of gene; Upstream/Downstream: the variations located within 1 kb upstream of a gene, and also within 1 kb downstream of another gene; ts: transition; tv: transversion.

### Selective sweep analysis

**FIGURE 2:**
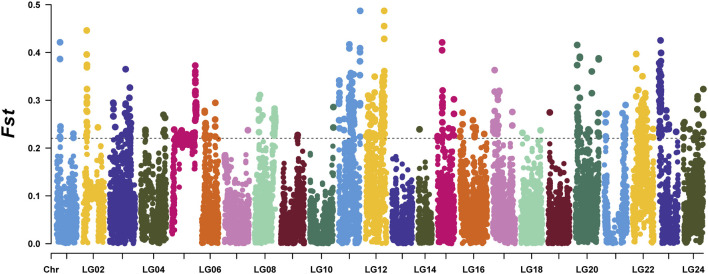
The distribution of *Fst* values between the mandarin fish which could be domesticated to accept artificial diets and those could not.

**FIGURE 3:**
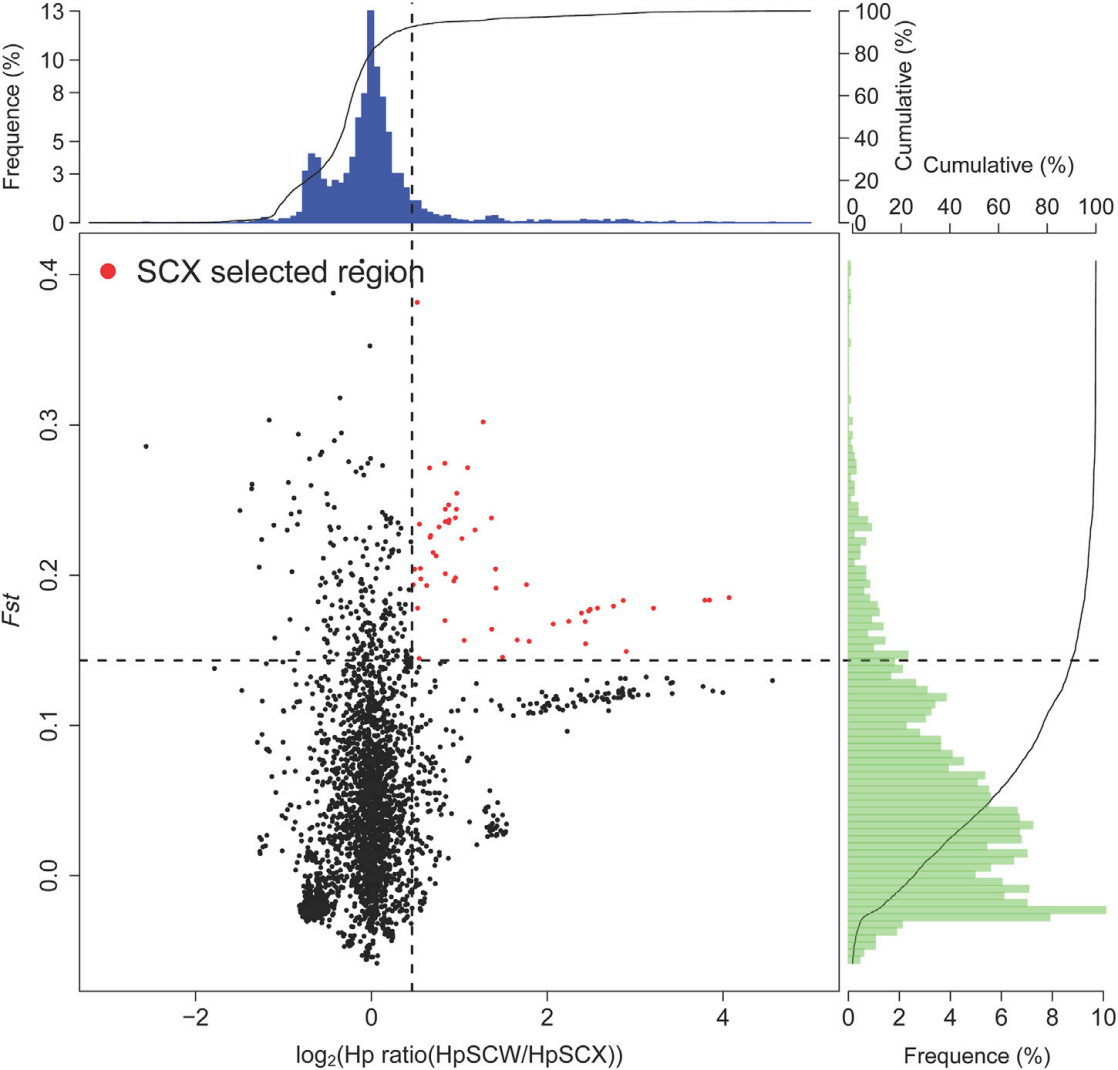
Selective sweep analysis. The regions were considered to be selected when both conditions are met: top 10% heterozygosity (*Hp*) and top 10% population differentiation coefficient (*Fst*).

**FIGURE 4:**
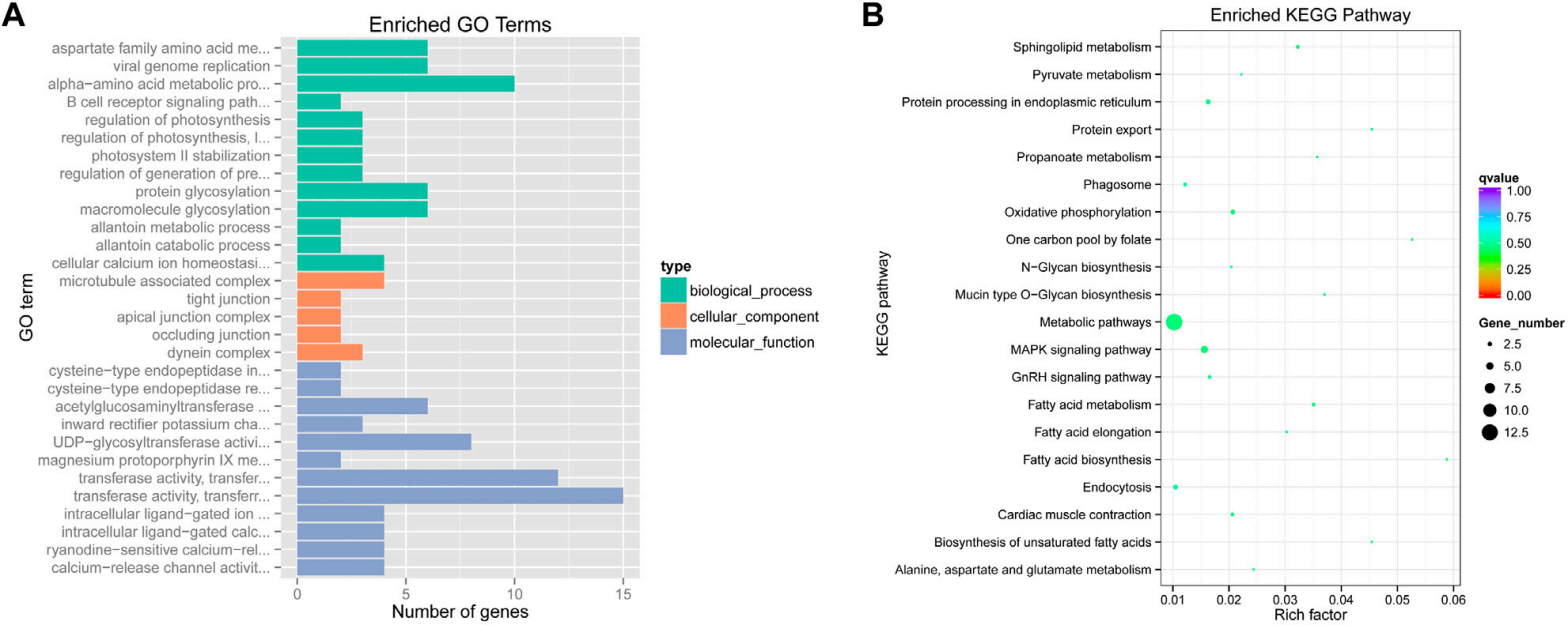
Functional enrichment analyses of potential selected genes. **(A)**. GO enrichment terms; **(B)**. KEGG enrichment pathways.

To identify selected regions for feeding habit domestication, selective sweep analysis based on heterozygosity (*Hp*) and population differentiation coefficient (*Fst*) was performed. A total of 15,023 windows were analyzed. The *Fst* values of 751 windows were above the threshold of 0.220615 (top 5% of *Fst* values) (Figure 2). 57 candidate windows were identified as putative selective regions for feeding habit domestication, involved in 149 genes (Figure 3). GO enrichment analysis revealed that these genes were significantly enriched in 162 GO items, including 88 GO terms in biological process, 11 GO terms in cellular component, and 63 GO terms in molecular function (Figure 4A, Supplementary Table S3). KEGG enrichment analysis revealed that these genes were significantly enriched in three KEGG pathways, including metabolic pathways, MAPK signaling pathway, and fatty acid metabolism (Figure 4B, Supplementary Table S4).

### Whole-genome bisulfite sequencing and mapping

In the present study, the hepatic DNA methylation of mandarin fish with different performance in accepting artificial diets were sequenced with whole-genome bisulfite sequencing (WGBS). A total of 225.34 Gb clean data were obtained. The Q20 (sequencing error rate <1%) were 97.75%–97.97% (Supplementary Table S5). 80.74%–91.22% read pairs could be mapped to our mandarin fish reference genome (version: sinChu7), and 76.93%–87.55% read pairs were uniquely mapped (Supplementary Table S6).

### DNA methylation patterns

The percentage of methylated cytosines to reference genomic cytosines were 6.96% and 6.44% in mandarin fish which could be domesticated to accept artificial diets and those could not. Methylated sites were counted in CG, CHG, and CHH contexts. Most methylated cytosines were in the CG dinucleotide context. The methylation levels of cytosines in CG context were 76.95% and 77.10% in mandarin fish which could be domesticated to accept artificial diets and those could not, respectively. The methylation levels of cytosines in CHG context were 0.60% and 0.55%, respectively. The methylation levels of cytosines in CHH context were 0.60% and 0.55%, respectively (Supplementary Table S7).

### Identification of differentially methylated regions and differentially methylated genes

**FIGURE 5:**
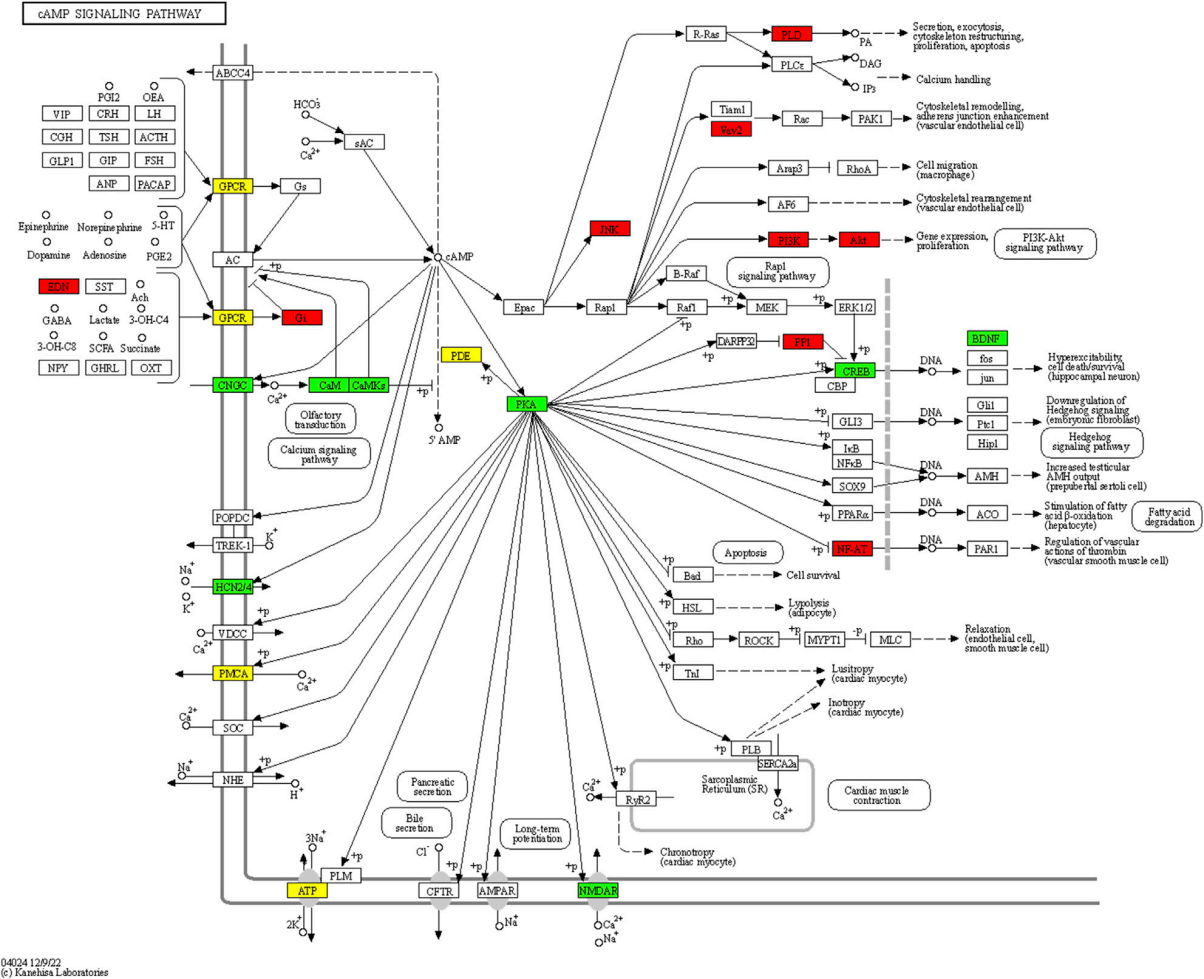
Differentially methylated genes in cAMP signaling pathway. Red: genes were hypermethylated; green: genes were hypomethylated; yellow: genes were both hypermethylated and hypomethylated.

5,976 differentially methylated regions (DMRs) were identified between mandarin fish with different performance in accepting artificial diets, of which 2,941 were hypermethylated and 3,035 were hypomethylated in mandarin fish which could be domesticated to accept artificial diets. A total of 3,522 differentially methylated genes were identified. GO enrichment analysis revealed that these genes were significantly enriched in 63 GO items, including 16 GO terms in biological process, 41 GO terms in cellular component, and 6 GO terms in molecular function (Supplementary Table 8). The representative KEGG pathway was cAMP signaling pathway, involved differentially methylated genes including cAMP-dependent protein kinase catalytic subunit beta (*prkacba*), cyclic AMP-responsive element-binding protein 5 (*creb5b*) and brain-derived neurotrophic factor (*bdnf*) (Figure 5). The region located within 2 kb upstream of *prkacba* gene, and the regions located in the 5′UTR introns of *creb5b* and *bdnf* genes were hypomethylated in mandarin fish which could be domesticated to accept artificial diets. In addition, the region located within 2 kb upstream of lipoprotein lipase gene (*lpl*) was hypomethylated in mandarin fish which could be domesticated to accept artificial diets.

## Discussion

To identify candidate genomic regions and genes for feeding habit domestication from live prey fish to artificial diets, the whole-genome resequencing was performed between mandarin fish which could be domesticated to accept artificial diets and those could not. Through the selective sweep analysis based on heterozygosity (*Hp*) and population differentiation coefficient (*Fst*), 57 candidate windows were identified as the putative selective regions for feeding habit domestication of mandarin fish, involved in 149 genes. These genes were related to memory, vision and olfaction function. Asparagine synthetase (ASNS) catalyses the synthesis of asparagine which is essential for brain development and function (Lomelino et al., 2017). Mutations in human *ASNS* gene caused a severe neurological condition, and the deficiency of Asns leads to brain structural abnormalities and memory deficits in mice (Ruzzo et al., 2013). In our previous study, comparative genomics analysis for four species of mandarin fish (Sinipercidae) indicated that *asns* gene was positively selected in *S. scherzeri*, which is the easiest to wean onto dead prey fish or artificial diets (He et al., 2020b), and the RNA-seq data also showed the lower mRNA expression level of *asns* in the mandarin fish which could accept dead prey fish than those could not (He et al., 2013). In the present study, *asns* gene was identified in the selected regions of mandarin fish which could be domesticated to accept artificial diets. And SNPs were identified within 2 kb upstream transcription start sites, 5′UTR, Exon 4 and introns of *asns* gene related to the artificial diet feeding habit. These results suggested that the *asns* gene might play an important role in the feeding habit domestication of mandarin fish. Microfibrillar associated protein 4 (MFAP4) is an extracellular matrix protein, contributing to innate immune defense in teleosts (Mohammadi et al., 2022), and *mfap4* was reported to be significantly up-regulated in the stomach of artificial diet domesticated mandarin fish (Shen et al., 2021). In the present study, *mfap4* gene was identified in the selected regions of mandarin fish which could be domesticated to accept artificial diets, suggesting that the *mfap4* gene might play an important role in the feeding habit domestication of mandarin fish through improving innate immunity.

Vision and olfaction are the most important sensory modalities, which are essential for the recognition of food in many fish species (DeBose and Nevitt, 2008; Konishi et al., 2022). Our previous study found that vision was the major sensory modality for mandarin fish to detect and catch prey (Liang et al., 2001). T-box transcription factor 2a (*tbx2a*) plays important roles in determination of photoreceptor fate (Angueyra et al., 2022) and expression regulation of opsins (Sandkam et al., 2020). TBC1 domain family member 20 (*TBC1D20*) gene encodes a key regulator of autophagosome maturation (Sidjanin et al., 2016). Mutations in human *TBC1D20* gene cause Warburg Micro syndrome, and one of the main symptoms is visual impairment (Liegel et al., 2013). *tbc1d20* was identified as a candidate adaptive allele in Caribbean pupfishes (Richards et al., 2021), and a candidate gene for domestication in rainbow trout (*Oncorhynchus mykiss*) (Cádiz et al., 2021). In the present study, *tbx2a* and *tbc1d20* were identified in the selected regions of mandarin fish which could be domesticated to accept artificial diets, suggesting that potential adaptive changes of vision might contribute to the feeding habit domestication from live prey fish to artificial diets in mandarin fish. Trace amine-associated receptor 13c (*taar13c*) is a member of olfactory receptor family, the encoded protein product was reported to have high-affinity for cadaverine (Hussain et al., 2013; Dieris et al., 2017). Cadaverine which is produced by decarboxylation of basic amino acids, is considered to be death-associated odor (Hussain et al., 2013). Cadaverine was detected during fishmeal processing, including raw materials and fishmeal (Nguyen et al., 2022). In the present study, *taar13c* was identified in the selected regions of mandarin fish which could be domesticated to accept artificial diets, indicating that they might have higher tolerance to cadaverine in the fishmeal, thus displaying higher acceptance in artificial diets.

Our previous studies indicated that DNA methylation might contribute to the feeding habit transformation in grass carp and mandarin fish (Cai et al., 2018; Dou et al., 2018). In the present study, the whole-genome bisulfite sequencing was performed between mandarin fish with different performance in accepting artificial diets. 5,976 differentially methylated regions were identified, involved in 3,522 genes. The representative KEGG pathway was cAMP signaling pathway, which could facilitate lipid metabolism in liver (Ravnskjaer et al., 2016). cAMP is an intracellular second messenger, and cAMP-dependent protein kinase (PKA) is essential for intracellular signal transduction (London and Stratakis, 2022). Cellular cAMP accumulation triggers the activation of PKA, then induces the phosphorylation of cAMP-response element binding protein (CREB), further activates the expression of BDNF (Wahlang et al., 2018). Brain-derived neurotrophic factor (BDNF) is a highly conserved member of neurotrophin family, playing important roles in synaptic plasticity and cognitive function (Lu et al., 2014). Except for the functions in nervous system, BDNF was reported to improve lipid and glucose metabolism in type-2-diabetic mice (Tsuchida et al., 2002). Impair of BDNF signaling might lead to metabolic syndrome (Marosi and Mattson, 2014). In the present study, differentially methylated regions were identified in genes involved in cAMP signaling pathway, including cAMP-dependent protein kinase catalytic subunit beta (*prkacba*), cyclic AMP-responsive element-binding protein 5 (*creb5*) and *bdnf*. The region located within 2 kb upstream of *prkacba* gene, and the regions located in the 5′UTR introns of *creb5b* and *bdnf* genes were hypomethylated in mandarin fish which could be domesticated to accept artificial diets. The hypomethylation of the regions located in upstream or 5′UTR intron of genes might increase the mRNA expression level of *prkacba*, *creb5* and *bdnf*, activating PKA-CREB-BDNF signaling, further improving lipid metabolism of mandarin fish which could accept artificial diets. This would be beneficial for mandarin fish to adapt to the artificial diets. In addition, the region located within 2 kb upstream of *lpl* gene was hypomethylated in mandarin fish which could be domesticated to accept artificial diets. Lipoprotein lipase (LPL) catalyzes the hydrolysis of triglycerides from circulating chylomicrons and very low density lipoproteins (Mead et al., 2002), playing important roles in lipid utilization and storage (Olivecrona, 2016). Loss-of-function mutations in LPL caused type I hyperlipoproteinemia in human, characterized by very severe hypertriglyceridemia (Caddeo et al., 2018). The DNA methylation change of placental *LPL* gene influenced fetal growth and fat accretion in childhood, which might further influence the metabolism in later life (Gagné-Ouellet et al., 2017). In the present study, due to the higher fat content of artificial diets than that of the live prey fish, the hypomethylation of the region located within 2 kb upstream of *lpl* gene might increase the mRNA expression of *lpl* gene, improving the lipid utilization. And our previous study found the differential metabolites related to lipid metabolism between mandarin fish which could be domesticated to accept artificial diets and those could not (He et al., 2021). These results suggested that lipid metabolism might play an important role in the adaption of artificial diets in mandarin fish.

In conclusion, we identified the selective genomic regions and genes, and the differentially methylated regions and genes for feeding habit domestication by whole-genome resequencing and bisulfite sequencing, respectively. These candidate genes could be critical for understanding the feeding habit domestication from live prey fish to artificial diets in mandarin fish, and further research is needed to clarify the roles of these candidate genes in feeding habit domestication.

## Data Availability

The datasets presented in this study can be found in online repositories. The names of the repository/repositories and accession number(s) can be found in the article/Supplementary Material. The resequencing data and WGBS data have been deposited into the NCBI Sequence Read Archive (SRA) database (BioProject: PRJNA895188; PRJNA896929, respectively).
